# Stem cell factor SALL4, a potential prognostic marker for myelodysplastic syndromes

**DOI:** 10.1186/1756-8722-6-73

**Published:** 2013-09-26

**Authors:** Fei Wang, Ye Guo, Qian Chen, Zhuo Yang, Ning Ning, Yujuan Zhang, Yonggang Xu, Xiaodong Xu, Chunrong Tong, Li Chai, Wei Cui

**Affiliations:** 1 Department of Clinical Laboratory, Peking Union Medical College Hospital, Peking Union Medical College and Chinese Academy of Medical Sciences, Beijing, China; 2 Department of Obstetrics and Gynecology, the First Affiliated Hospital, Harbin Medical University, Harbin, China; 3 Department of Hematology, Xiyuan Hospital of China Academy of Chinese Medical Sciences, Beijing, China; 4 Department of Hematology, Beijing Hospital, Beijing, China; 5 Department of Hematology, Beijing Daopei Hospital, Beijing, China; 6 The Department of Pathology, Brigham and Women’s Hospital, Harvard Medical School, Boston, USA

**Keywords:** SALL4, Myelodysplastic syndromes, Prognosis

## Abstract

**Background:**

Myelodysplastic syndromes (MDS) are a group of heterogeneous diseases with variable clinical course. Predicting disease progression is difficult due to lack of specific molecular marker(s). SALL4 plays important roles in normal hematopoiesis and leukemogenesis. SALL4 transgenic mice develop MDS prior to acute myeloid leukemia (AML) transformation. However, the role of SALL4 in human MDS has not been extensively investigated. In this study, we evaluate the diagnostic/prognostic value of SALL4 in MDS by examining its expression levels in a cohort of MDS patients.

**Methods:**

Fifty-five newly diagnosed MDS, twenty MDS-AML, and sixteen post-treatment MDS patients were selected for our study along with ten healthy donors.

**Results:**

We demonstrated that SALL4 was over-expressed in MDS patients and proportionally increased in MDS patients with high grade/IPSS scores. This expression pattern was similar to that of Bmi-1, an important marker in predicting MDS/AML progression. In addition, the level of SALL4 was positively correlated with increased blast counts, high-risk keryotypes and increased significantly in MDS-AML transformation. Furthermore, higher level of SALL4 expression was associated with worse survival rates and SALL4 level decreased following effective therapy.

**Conclusions:**

To the best of our knowledge, this is the largest series and the first to report the expression pattern of SALL4 in detail in various subtypes of MDS in comparison to that of Bmi-1. We conclude that SALL4 is a potential molecular marker in predicting the prognosis of MDS.

## Background

Myelodysplastic syndromes (MDS) are clonal hematopoietic stem cell (HSC) disorders characterized by refractory cytopenias with dysplasia as a result of ineffective hematopoiesis [1]. Approximately 30 percent of MDS cases progress to acute myeloid leukemia (AML) [2]. Excess blasts are the strongest predictors for poor outcome and are associated with disease progression [3]. Because of its heterogeneity and lack of molecular markers that effectively monitor disease progression, clinical management of MDS patients is challenging. The International Prognostic Scoring System (IPSS) is widely used to predict the prognosis of MDS [3,4], however, the prognostic information it provides is not always coincident with clinical outcome [5]. Recently, a few genetic mutations and abnormal expressions were reported to be involved in MDS pathogenesis and showed potential clinical value [6-11].

SALL4 is an important embryonic stem cell (ESC) factor that is also involved in normal hematopoiesis, leukemogenesis [12-20], hepatocellular carcinoma [21-23] and infantile hemangiomas [24]. Due to its essential role for the maintenance of pluripotent and self-renew of hematopoietic stem cell, SALL4 exhibits potential value in expansion of hematopoietic stem cell in vitro [25-27]. We have previously reported that SALL4 was constitutively expressed in human AML and SALL4 transgenic mice developed MDS–like features and subsequently AML [18]. We have also demonstrated recently that the expression level of SALL4 was related to AML treatment status [16]. SALL4 had the highest expression level in untreated AML patients, gradually decreased expression levels in partial remission (PR) and in complete remission (CR) patients. Over-expression of SALL4 was also associated with drug resistance. SALL4 expression levels declined during the treatment process in the drug responsive group and increased in the drug resistant group.

Bmi-1 is a member of the polycomb repression complex, and is required for the maintenance and self-renewal of normal and leukemic stem and progenitor cells [28-30]. Our previous study linked Bmi-1 and SALL4, showing Bmi-1 as a direct target gene of SALL4 in normal hematopoietic and leukemic cells [31]. SALL4 could bind to the Bmi-1 promoter; furthermore, induction of SALL4 expression was associated with increased levels of histone H3–K4 and H3–K79 methylation in the Bmi-1 promoter. Others [9] showed that Bmi-1 expression in CD34+ cells was high in refractory anemia with excess blasts (RAEB), RAEB in transformation (RAEB-T), and MDS-AML. The expression level of Bmi-1 was correlated with IPSS score, suggesting that Bmi-1 can be a potential marker for predicting the prognosis of MDS.

Since MDS is a pre-leukemic stage of AML [32] and the expression pattern of SALL4 in human MDS hasn’t been intensively investigated, in this study we evaluate the expression of SALL4 in various MDS patients compared to that of Bmi-1 to explore the potential clinical prognostic value of SALL4 in MDS.

## Results

### The expression of SALL4 was increased in MDS patients and correlated with disease progression and treatment status

**Figure 1:**
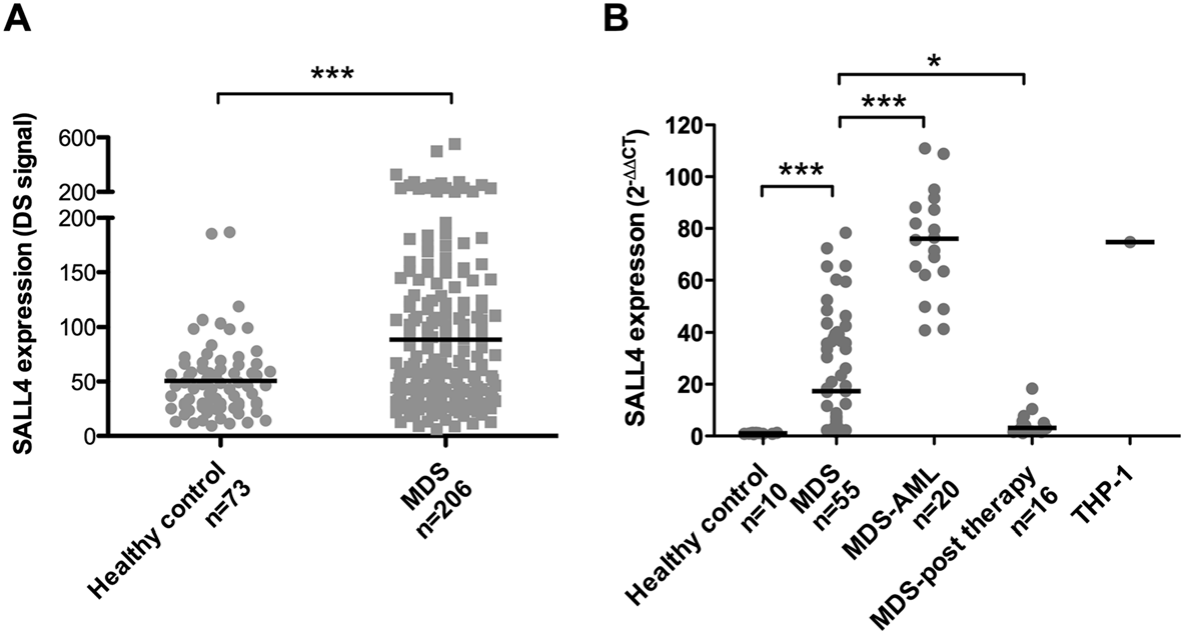
**SALL4 expression was increased in MDS patients and correlated with disease progression and treatment status. (A)** SALL4 expression levels by microarray in 206 MDS cohort (GSE13159). The data was presented as log-transformed scaled signal (DS signal). **(B)** SALL4 expression in the healthy control, MDS, MDS-AML, MDS-post treatment and THP-1 positive control analyzed by qRT-PCR. * *P*-value<0.05, ***p-value<0.001. The lines indicate mean value of each group.

We first evaluated the expression of SALL4 in primary MDS patients by analyzing MDS gene expression profiles from the public database GSE13159 [33]*.* The data was presented as log-transformed scaled signal (DS signal) [34]. We noticed that SALL4 expression measured by microarray was higher (*p*<0.001) in MDS patients (88.49±77.57, n=206) than that from normal controls (50.54±34.13, n=73) (Figure 1A).

We next chose 55 newly diagnosed MDS patients, 20 MDS-AML patients and 16 post-treatment MDS patients for our study and the clinicopathological profile of these patients was summarized in Table 1. The expression level of each gene was presented as 2^-ΔΔCT^ relative quantitative values. Our results demonstrated that SALL4 mRNA expression level in MDS patients (23.88±21.59, n=55) was significantly higher (p<0.001) than that from the healthy control group (1.02±0.19, n=10). Furthermore, SALL4 expression increased significantly (p<0.001) in MDS-AML (AML patients with history of MDS) patients (74.35±20.00, n=20), but deceased (p<0.05) in post-treatment MDS patients (4.66±4.43, n=16) (Figure 1B).

**Table 1:** Characteristics of patients with MDS, MDS-AML and MDS post-treatment

		**MDS**				**MDS-AML**	**MDS post-treatment**
		**RA**	**RCMD**	**RAEB-1**	**RAEB-2**		
**Patients (N)**		18	10	14	13	20	16
**Gender**							
	Male	5	6	9	7	10	10
	Female	13	4	5	6	10	6
**Age (year)**							
	Median	36	48.5	69	65	68.5	53
	Range	22-72	20-77	52-81	25-82	28-82	22-74
**BM Blast (%)**							
	Median	1	1.75	6.5	15.5	55	0.75
	Range	0-3.5	0.5-4.5	5-8.5	11-19.5	23-98	0-7.5
Karyotype (N)*							
	Good	14	7	7	4	5	13
	Intermediate	4	2	2	0	3	3
	Poor	0	1	5	9	12	0
**IPSS score**							
	Median	0.5	0.5	1.25	3	––	––
	Range	0-1	0-1.5	1-2	2-3	––	––

*Karyotype: Good=normal, -Y alone, del(5q) alone, del(20q) alone; Poor=complex (≥3 abnormalities) or chromosome 7 abnormalies; Intermediate=other abnormalities.

### SALL4 and Bmi-1 shared a similar expression pattern in human MDS with both expressions increased in high-grade morphology and high IPSS score cases

**Figure 2:**
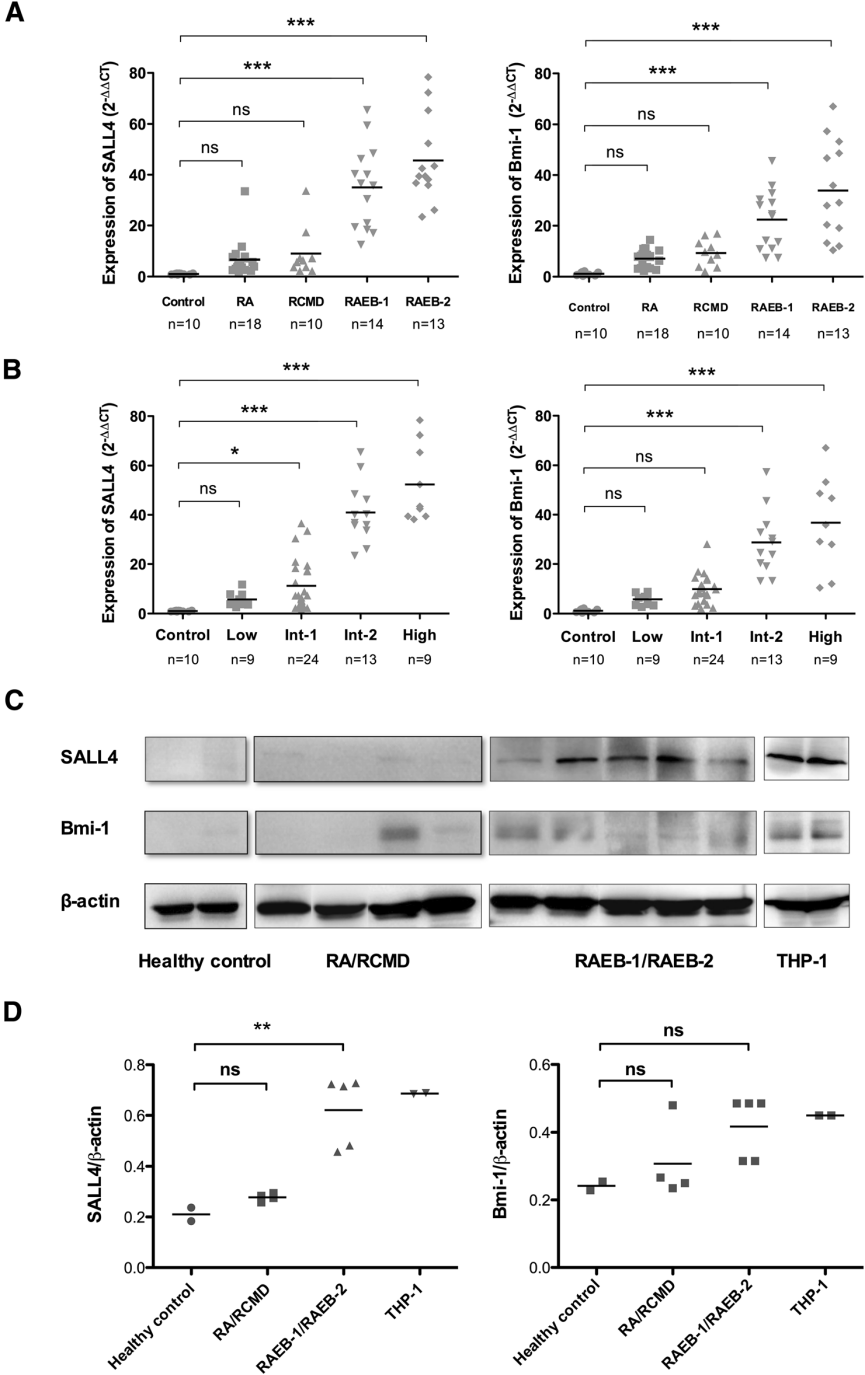
**SALL4 and Bmi-1 shared a similar expression pattern in MDS: both were increased in high-grade morphology and high IPSS score cases. (A)** SALL4 and Bmi-1 expression in subtypes of MDS according to WHO classification. **(B)** SALL4 and Bmi-1 expression in different IPSS risk groups. **(C)** Representative SALL4 and Bmi-1 protein expression analyzed by western blot. The protein expression of SALL4 in RAEB-1/RAEB-2 was higher than that of healthy control. Bmi-1 protein expression followed a similar trend. THP-1 cell line was used as a positive control. **(D)** Semi-quantitatively analysis demonstrated that this trend was statistically significant for SALL4 but not for Bmi-1. * *P*-value<0.05, **p-value<0.01, ***p-value<0.001, ns: no significance. The lines indicate mean value of each group.

Because MDS is a group of heterogeneous diseases, different subtypes and risk groups exhibit great heterogeneities. We next evaluated SALL4 expression in different WHO morphology and IPSS risk groups and compared expression with that of Bmi-1. Between different MDS subtypes by WHO classification, the expression of SALL4 was significantly higher (*p*<0.001) in RAEB-1 (35.06±16.32, n=14) and RAEB-2 (45.66±16.87, n=13) subtypes when compared with healthy control, but not refractory cytopenia (RA) (6.62±7.21, n=18) or refractory cytopenia with multilineage dysplasia (RCMD) (8.95±9.72, n=10). Similarly, higher (*p*<0.001) expression of Bmi-1 was also preferentially seen in RAEB-1 (22.47±11.89, n=14) and RAEB-2 (33.92±19.00, n=13) (Figure 2A). Likewise, between different risk groups of MDS based on IPSS, Int-1 (11.19±10.35, n=25), Int-2 (40.97±12.36, n=12) and High risk groups (52.37±15.65, n=9) had higher (*p*<0.05) SALL4 expressions than healthy control. As for Bmi-1 expression, Int-2 (28.77±13.00, n=12) and High risk group (36.77±18.97, n=9) showed significantly higher (*p*<0.001) levels than healthy control (Figure 2B). Furthermore, we performed western blots to see SALL4 and Bmi-1 expression on protein level and found that the protein expression of these two genes followed the same trend (Figure 2C). However, semi-quantitatively analysis demonstrated that this trend was statistically significant for SALL4 (p<0.01) but not Bmi-1 (p>0.05) (Figure 2D).

### SALL4 expression was correlated with blast counts, IPSS score, Bmi-1 and poor cytogenetic characters

**Figure 3:**
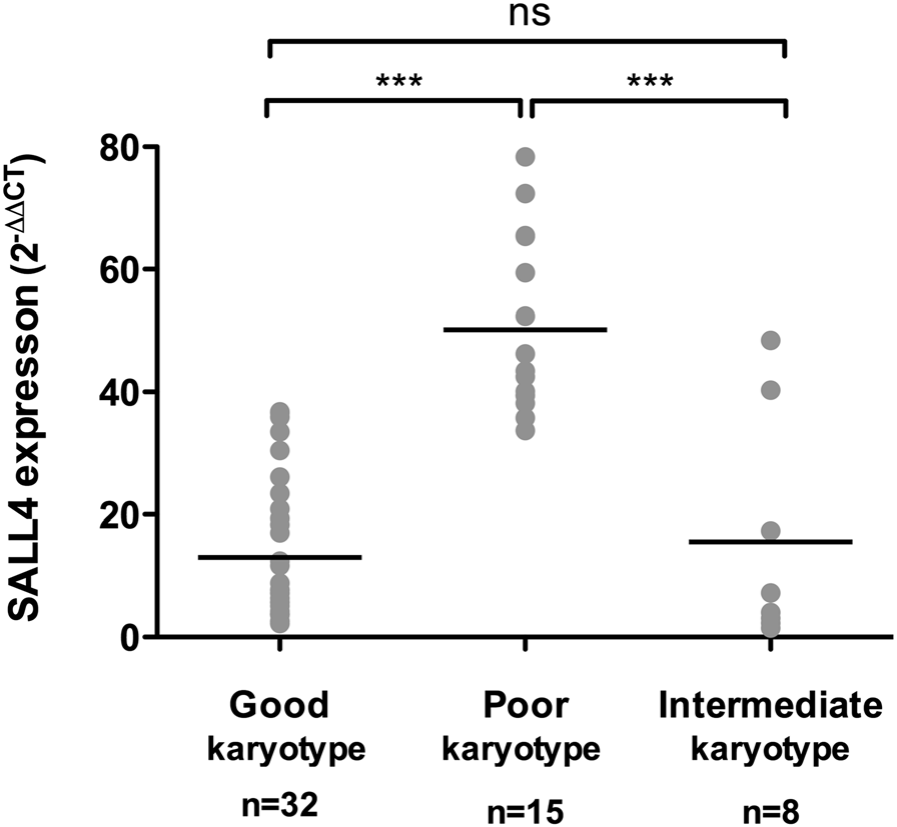
**SALL4 expression was correlated with cytogenetic characters.** SALL4 expression in MDS patients with high-risk karyotypes was significantly higher than patients with intermediate and low karyotypes, however there was no significant difference between patients with intermediate and low karyotypes. The risk classification of karyotypes was based on IPSS. ***P-value<0.001, ns: no significance. The lines indicate mean value of each group.

To investigate the potential value of SALL4 in the clinical diagnostic application of MDS patients, we next conducted Spearman’s correlation analysis on the mRNA expression of SALL4 and clinical parameters. Our results showed that SALL4 was positively correlated with bone marrow blast counts, IPSS score and expression level of Bmi-1 (Table 2). In addition, we observed that SALL4 expression was higher (*p*<0.001) in MDS patients with high risk (50.14±14.44, n=15) karyotypes according to IPSS, including complex karyotypes (n=14) and chromosome 7 abnormalities (n=1), but lower (*p*<0.001) in patients with intermediate (15.53±18.62, n=8) and low risk (13.02±11.51, n=32) karyotypes (Figure 3).

**Table 2:** Spearman correlation analysis between SALL4 and clinical parameters in MDS patients

**Clinical parameters**	**Correlation coefficient (r**_**s**_**)**	***p *- value**
SALL4 vs. BM blast (%)	0.802	<0.001
SALL4 vs. IPSS score	0.808	<0.001
SALL4 vs. Bmi-1	0.790	<0.001

### MDS patients with high SALL4 expression showed disease progression and worse survival

**Figure 4:**
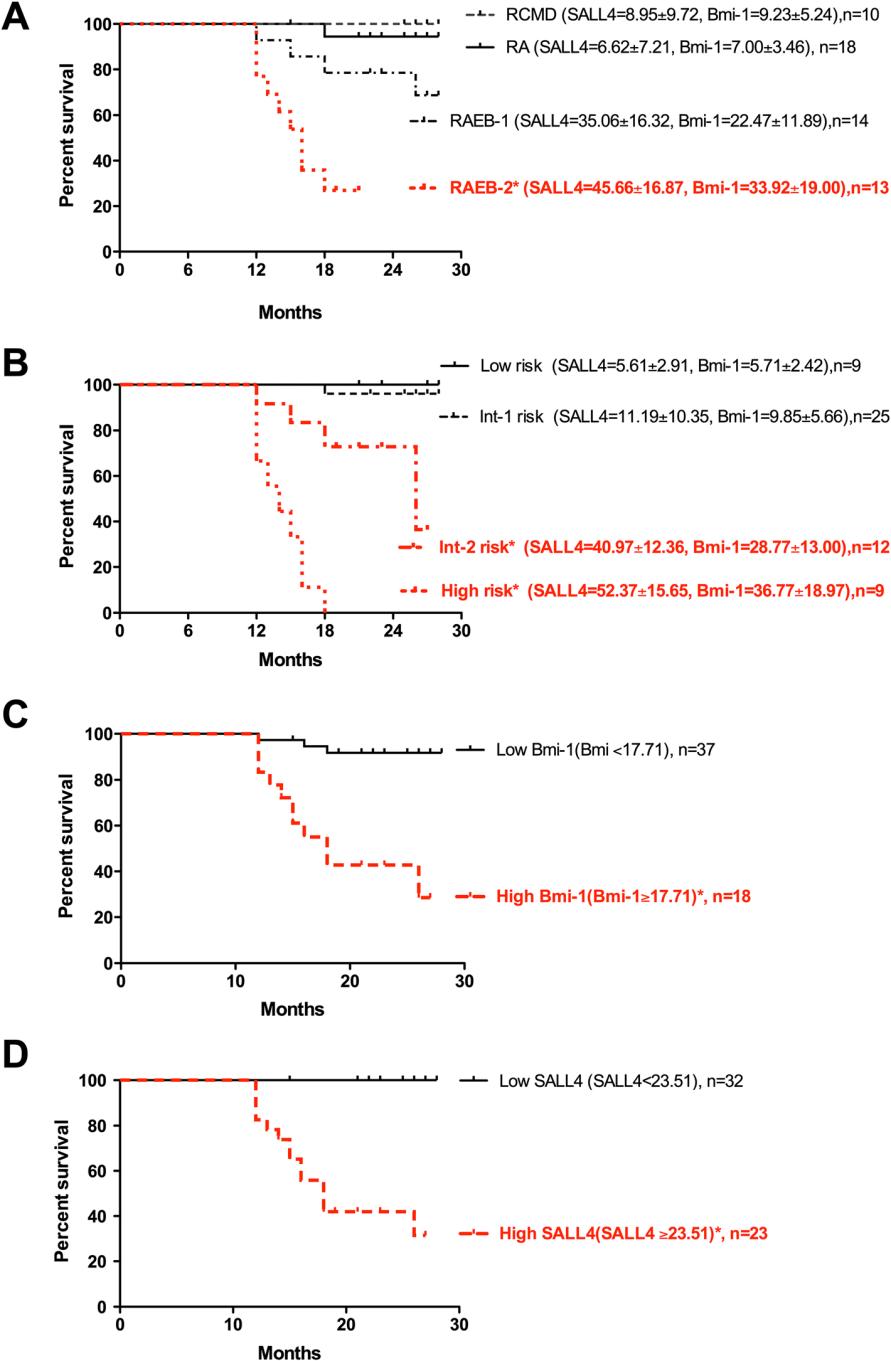
**MDS patients with high SALL4 expression had worse survival rates. (A)** Kaplan-Meier survival curves for MDS patients based on WHO subtypes. The RAEB-2 group with higher SALL4 and Bmi-1 expression levels showed a worse survival rate. **(B)** Kaplan-Meier survival curves for MDS patients based on IPSS risk groups. Int-2 and High risk groups had worse survival rates than Int-1 and Low risk groups. Correspondingly, SALL4 and Bmi-1 expression levels were also higher in Int-2 and High risk groups. **(C)** Kaplan-Meier survival curves for MDS patients based on Bmi-1 expression level. **(D)** Kaplan-Meier survival curves for MDS patients based on SALL4 expression level. *P-value<0.05 analyzed by log-rank test. The expression data on SALL4 and Bim-1 were presented as mean±SD.

During our over 2 years’ follow-up, 11 of 27 (40.74%) RAEB-1/RAEB-2 patients experience disease progression. SALL4 expression of these cases (54.27±14.71, n=11) was significantly higher (p<0.01) than those without disease progression (30.47±10.89, n=16). 14 patients died of MDS or AML progressed from MDS. Since both IPSS and WHO have been used to guide clinical diagnosis and management of MDS patients, we first conducted Kaplan-Meier survival analysis for MDS patients based on WHO subtypes and IPSS risk groups. Based on WHO subtypes, the RAEB-2 group that showed high SALL4 and Bmi-1 expression had a worse (*p*<0.01) survival rate than other subtypes (Figure 4A). Based on IPSS risk groups, Int-2 and High risk patient groups whose SALL4 and Bmi-1 expression levels were higher than that from Int-1 and Low risk patient groups exhibited a worse (*p*<0.01) survival rate (Figure 4B). Intriguingly, we noted that among the Int-2 risk group, patients who passed away during the follow up (53.42±11.25, 4 of 12) had higher (*p*=0.0056) SALL4 expression than those who were still alive at the end of the study (34.75±7.82, 8 of 12). This trend was also observed for Bmi-1 expression. Patients who passed away in the Int-2 risk group had higher Bmi-1 expression (30.91±4.78, 4 of 12), while patients who were still alive had lower Bmi-1 expression (27.69±15.87, 8 of 12). However, this difference was not significant (*p*=0.7058).

We further conducted Kaplan-Meier survival analysis for MDS patients based on Bmi-1 or SALL4 expression level (The group with high Bmi-1 or SALL4 expression had Bmi-1 or SALL4 mRNA expression that was equal to or exceeded the mean value detected by qRT-PCR, where as the group with low Bmi-1 or SALL4 expression had Bmi-1 or SALL4 mRNA expression that was lower than the mean value). MDS patients with high Bmi-1 or SALL4 expression levels showed a worse (*p*<0.01) survival rate than low Bmi-1 or SALL4 group (Figure 4C-D). These results suggest that SALL4 and/or Bmi-1 expression could be used as an additional confirmation/refinement for the WHO/IPSS system or even as single or combined molecular tests to help predict the prognosis of MDS. More detailed analysis, however, showed minor differences between the Bmi-1 and SALL4 expression in the survival rate of MDS patients. There was no patient death with low SALL4 expression (Figure 4D), but 3 patients died with low Bmi-1 expression (Figure 4C). This result suggested that SALL4 might be a better molecular marker than Bmi-1, in predicting MDS patient prognosis; however a large cohort study is needed to confirm this result.

## Discussion

In this study we have examined the expression levels of stem cell factor SALL4 in MDS patients from various MDS morphology subtypes and IPSS risk groups in comparison to Bmi-1 expression levels in these patients. Our study showed that SALL4 expression was high in newly diagnosed MDS patients and increased significantly in MDS-AML patients, but deceased in post-treatment MDS patients. Higher SALL4 expression was seen in MDS patients with high grade/IPSS scores, an expression pattern similar to that of Bmi-1. We have also evaluated the correlation between SALL4 expression and several clinical parameters such as bone marrow blast count, karyotype and IPSS scores. This cohort of MDS patients was followed up with for about two years. We noticed that MDS patients with high SALL4 expression had a worse survival rate. When taken together, our study results suggest that SALL4 can be used as a potential new molecular marker in predicting the prognosis of MDS. To our knowledge, this is the first study to analyze SALL4 prognostic value in MDS patients.

Recently another study reported a significant positive correlation between the level of SALL4 transcript and the status of SALL4 hypomethylation in MDS patients [35]. They also found that the patients in higher IPSS risk groups (Int-2/High) and WHO classifications had significantly higher incidence of SALL4 hypomethylation than those in low risk groups (Low/Int-1) and mild WHO classifications. It remains to be determined whether hypomethylation is a mechanism for aberrant high SALL4 expression in MDS patients with higher IPSS risks and WHO subtypes.

One unique feature of our MDS patient cohort is that the median age of these patients is only 56 (from 20 to 82), which is younger than most reported median ages for MDS patients. This may be due to the fact that in China, most patients pay for their own medical care expenses, and younger patients will seek medical care more readily than older patients due to underlying social-economical reasons.

In our study, SALL4 expression is related to several clinical parameters. For example, bone marrow blast count, an important factor that contributes to WHO categories and IPSS risk groups, was positively related to SALL4 expression level. This finding was in agreement with previous studies that demonstrated that SALL4 was expressed preferentially in normal purified CD34^+^ hematopoietic stem/progenitor cells as well as in leukemic blasts in AML and B-cell lymphoblastic leukemia/lymphomas [13-15,18]. This also explained why SALL4 expression in MDS was lower than that from MDS-AML as mentioned above. In this study, we have also evaluated SALL4 expression and its correlation with cytogenetic abnormalities. Cytogenetic abnormalities have been found at diagnosis in 20-70% of MDS patients, especially in RAEB-1/RAEB-2 cases [1,36], and are critical in evaluating the outcome of patients with MDS. In our study, we observed that SALL4 was highly expressed in MDS patients with complex karyotypes and chromosome 7 abnormalities. Further study is needed to investigate whether high SALL4 expression contributes to chromosome disability or is the result of complex phenotype.

Our survival analysis demonstrated that MDS patients with higher SALL4 expression had a worse survival rate. Intriguingly, in our study, most of the MDS patients that died were from IPSS High/Int-2 risk or WHO RAEB-1/2 groups except one case, which was classified as WHO RA subtype with an IPSS score of Int-1 risk group. However, the SALL4 expression of this patient was as high as 33.50 at the time of diagnosis. Because the patient was diagnosed with RA/Int-1 risk, only supportive treatment was provided. This patient passed away 18 months afterward. Though this is only one case and a larger study size is needed, it seems to suggest that SALL4 expression could be used at least as an additional confirmation/refinement for the IPSS/WHO system to help predict the prognosis of MDS, and to confirm high-risk patients can get proper treatment as soon as possible.

## Conclusions

In summary, we are the first to report the expression pattern of SALL4, an embryonic stem cell factor and a leukemic survival factor, in various morphological subtypes and risk groups of MDS along with a comparison to Bmi-1. Our study suggests that SALL4 can be used as a potential molecular marker in predicting poor prognosis of MDS. Furthermore, qRT-PCR, a simple test to evaluate SALL4 expression, is a good and measurable proxy for bone marrow blast cell count which is notoriously difficult to standardize. Though a future prospective study with a large group of patients is required to further evaluate our observations, our results suggest that SALL4 expression level can potentially be used to guide decision making in the treatment of MDS patients.

## Methods

### Cells

THP-1 Human Monocytic Leukemia Cells (Cell Center, Institute of Basic Medical Sciences, Chinese Academy of Science) were cultured in RPMI 1640 supplemented with 10% fetal bovine serum.

### Patients and control samples

Bone marrow samples from 55 newly diagnosed MDS patients, 20 MDS-AML patients, 16 post-treatment MDS patients and 10 healthy donors were collected in Beijing from December 2009 to December 2011. This study was approved by the institutional review board of Peking Union Medical College Hospital. The diagnosis of MDS was based on the 2008 World Health Organization (WHO) Classification [37]. Patients with low or Int-1 IPSS risk received clinical monitoring and relevant supportive care. In general, patients with symptomatic anemia or severe thrombocytopia were treated with relative transfusion. Recombinant human Epo with or without recombinant human granulocyte colony stimulating factor (G-CSF) were used for patients with serum erythropoietin (EPO) levels ≤500 mU/ml. Azacytidine or decitabine were used for patients with thrombocytopenia or neutropenia. For patients with del (5q) chromosomal abnormalities, Lenadomide was used for treatment. Int-2 or high risk patients received high intensive therapy including intensive chemotherapy (such as azacytidine and decitabine) or allogeneic hematopoietic stem cell transplantation (HSCT) if available. For patients who were not suitable (e.g., patient’s age, performance status and patient’s preference) for intensive therapy, relative supportive care was given. Bone marrow samples from post-treatment MDS patients were collected after the patients completed at least two-course treatment.

The 206-MDS-cohort was downloaded from Gene Expression Omnibus (GEO) dataset [38] under series accession number GSE13159. The data in this microarray was presented as log-transformed scaled signal (DS signal) [34].

### Isolation of bone marrow nucleated cells

Red blood cell lysis buffer was used to isolate nucleated cells from bone marrow samples according to a standard procedure. The isolated nucleated cells were then divided into two parts, one of which was conducted with Trizol reagent (Invitrogen) for RNA analysis, the other of which was processed with Cell Lysis Buffer (Beyotime Biotchnology, China) for further protein analysis.

### Quantitative-reverse-transcription-polymerase-chain-reaction (qRT-PCR)

Total RNA was extracted with Trizol reagent (Invitrogen) and treated with DNase. Reverse transcription and PCR amplification were performed according to manufacturer’s instruction (DRR036 and DRR091, TaKaRa). All of the reactions were run in triplicate. The expression level of each gene was normalized to the housekeeping gene β-actin and analyzed by 2^-ΔΔCT^ relative quantitative method. The sequences of primers were as follows: SALL4 (amplicon length, 68bp; targated Exon2 of human SALL4), forward primer 5′-TGCAGCAGTTGGTGGAGAAC-3′, reverse primer 5′-TCGGTGGCAAATGAGACATTC-3′ [18]; Bmi-1 (amplicon length, 198 bp), forward primer 5′-TGGACTGACAAATGCTGGAGA-3′, reverse primer 5′-GAAGATTGGTGGTTACCGCTG-3′; β-actin (amplicon length, 124 bp), forward primer 5′-TGTACGCCAACACAGTGCTG-3′, reverse primer 5′-TCAGGAGGAGCAATGATCTTG-3′.

### Western blot

Western blot was performed according to standard techniques. The following antibodies were used: SALL4 (ab29112, Abcam), Bmi-1 (05–637, Mlillipore), and β-actin (TA-09, ZSGB-BIO, China). The dilution ratio of SALL4 was 1:1000, Bmi-1 1:1000 and β-actin 1:2000.

### Statistical analyses

Statistical analyses were performed using SPSS software package Version 12.0. *P*-values of <0.05 were considered statistically significant. Data were presented as means ± standard deviation. Unpaired t-test or Mann–Whitney test was used to study difference between two groups. One-Way ANOVA or Kruskal-Wallis test were applied to study differences between more than two groups. When significance was found, Bonferroni’s multiple comparison test or Dunnett’s multiple comparison test was used as a post hoc test. Correlation analysis was performed using the Spearman’s rank correlation coefficient r_s_. Survival curves were constructed using the Kaplan-Meier method and compared using the log-rank test.

## Abbreviations

MDS: Myelodysplastic syndromes; AML: Acute myeloid leukemia; IPSS: International prognostic scoring system; RA: Refractory anemia; RCMD: Refractory cytopenia with multilineage dysplasia; RAEB: Refractory anemia with excess blasts; RAEB-t: RAEB in transformation.

## Competing interests

The authors declare that they have no competing interests.

## Authors’ contributions

FW designed research, performed experiments, analyzed data and wrote the manuscript; YG, QC, ZY, NN and YZ performed part of the experiments; YX, XX, and CT provided the samples and helped with data collection. WC and LC designed the research, supervised the experiments, and edited the manuscript. All authors read and approved the final manuscript.
